# Cardiovascular risk assessment and treatment in chronic inflammatory disorders in primary care

**DOI:** 10.1136/heartjnl-2016-310111

**Published:** 2016-08-17

**Authors:** G Emanuel, J Charlton, M Ashworth, M C Gulliford, A Dregan

**Affiliations:** 1 Department of Primary Care and Public Health, King's College London, London, UK; 2 National Institute for Health Research Biomedical Research Centre at Guy's and St Thomas’ National Health Service Foundation Trust, Kings’ College London, London UK

## Abstract

**Objective:**

To compare differences in cardiovascular (CV) risk factors assessment and management among patients with rheumatoid arthritis (RA) and inflammatory bowel disease (IBD) with that of matched controls.

**Methods:**

A matched cohort study was conducted using primary care electronic health records for one London borough. All patients diagnosed with RA or IBD, and matched controls registered with local general practices on 12th of January 2014 were identified. The study compared assessment and treatment of CV risk factors (blood pressure, body mass index, cholesterol and smoking) in the year before, the year after, and 5 years after RA and IBD diagnosis.

**Results:**

A total of 1121 patients with RA and 1875 patients with IBD were identified and matched with 4282 and, respectively, 7803 controls. Patients with RA were 25% (incidence rate ratio, 1.25, 95% CI 1.12 to 1.35) more likely to have a CV risk factor measured compared with matched controls. The difference declined to 8% (1.08, 1.04 to 1.14) over 5 years of follow-up. The corresponding figures for IBD were 26% (1.26, 1.16 to 1.38) and 10% (1.10, 1.05 to 1.15). Patients with RA showed higher antihypertensive prescription rates during 5 years of follow-up (OR, 1.37, 95% CI 1.14 to 1.65) and patients with IBD showed higher statin prescription rates in the year preceding diagnosis (2.30, 1.20 to 4.42). Incomplete CV risk assessment meant that QRISK scores could be calculated for less than a fifth (17%) and clinical recording of CV disease (CVD) risk scores among patients with RA and IBD was 11% and 6%, respectively.

**Conclusions:**

The assessment and treatment of vascular risk in patients with RA and IBD in primary care is suboptimal, particularly with reference to CVD risk score calculation.

Chronic inflammatory disorders are increasingly acknowledged to be associated with heightened risk of cardiovascular disease (CVD) comorbidity, the leading cause of death worldwide.^1–5^ A recent study with primary care data documented increased risk of stroke, type 2 diabetes and coronary heart disease among several inflammatory disorders, including rheumatoid arthritis (RA) and ulcerative colitis.^5^ The increasing recognition for heightened CVD risk among chronic inflammatory patients has led to the development of clinical recommendations to facilitate the prevention of inflammation-related CVD risk. These guidelines recommend the screening of inflammatory patients for vascular risk factors including blood pressure (BP), cholesterol, smoking, body mass index (BMI) and the use of a validated CVD risk score to predict CVD risk.^6^ The extent to which these recommendations are implemented into routine care is, however, poorly understood. Earlier evidence from rheumatology clinic settings suggested deficient vascular risk assessment practices.^7–9^ This evidence is less available within a primary care context—the main setting for CVD risk assessment and treatment in the UK. Recently, Alemao *et al*^10^ found no differences in the assessment and treatment of CV risk factors among patients with RA in a large primary care database. The extent to which national recommendations are implemented at a more local level or across different inflammatory disorders is less explored. The main aim of the present study was to identify the extent to which primary care practitioners from one of the most deprived and ethnically diverse areas of the UK implemented recommended guidelines for CV risk factors assessment and treatment among patients with RA and inflammatory bowel disease (IBD).

## Methods

### Data

The data for the present study were based on the Lambeth DataNet, a patient-level primary care database containing electronic health records data for over 350 000 patients living in one London borough (Lambeth), including their demographics, clinical diagnoses, specialist services referral, laboratory tests and treatment. Lambeth is among the most ethnically diverse borough in the UK and the ninth most deprived borough in London, with 36.7% of its population living in the most deprived lower super output areas (LSOAs) in England. The study population was selected using Read medical codes and included all patients diagnosed with RA or IBD (including ulcerative colitis and Crohn's disease) after current registration date (the date when the patient was registered uninterruptedly with the practice). Patients with a diagnosis of the selected disorders were matched on age, gender and general practice with up to four controls randomly sampled from all patients who were disease-free. The controls were also required to be registered at the case index date. The index date for controls was determined as the closest observation to the case index date.

### Study measurements

The management of traditional CV risk factors and CVD risk score was evaluated as the percentage of patients screened for at each CV risk factor and the percentage of patients prescribed antihypertensive and lipid-lowering therapies at three different time points: the 12 months before the RA and IBD index date, the 12 months following the index date, and within 5 years following the index date. The study also evaluated the percentage of patients measured for three or four, as well as for all four CV risk factors at the specified time periods. We also calculated the proportion of patients that were hypertensive (systolic blood pressure (SBP)/diastolic blood pressure ≥140/90 mm Hg, or were on the hypertension register), hypercholesterolemic (total cholesterol ≥5 mmol/L), obese (BMI>29) or current smokers at the specified time points. Additionally, we estimated the percentage of patients with a recorded CVD risk score, as well as the percentage of patients with available data to calculate a QRISK score—a prediction algorithm to assess CVD risk based on the QRESEARCH database.^11^ BP, total cholesterol, BMI and smoking data from medical records were analysed in patients with RA/IBD and their matched controls. CVD risk assessment was defined as the recording of a CV risk score (including Framingham Risk Score, Joint British Societies risk score, QRISK score). All CV risk factors and relevant therapies were operationalised as binary variables—yes (1) if the risk factor was measured and no (0) if the risk factor was not recorded during the specified time points.

### Covariates

Several covariates measured at baseline were considered including patients' gender (male/females) and age at index date (the case index was used for matched controls). Ethnicity was self-reported by patients, and classified patients into White, Black, Asian, Mixed and Other. Ethnicity was categorised as missing if recorded as refused or not recorded. Deprivation was assessed by mapping patient postcodes to the lower super output area (LSOA) and assigning the index of multiple deprivation (IMD 07) score for that area. Scores were then grouped into five quintiles, from most to least deprived. The study also considered baseline differences between patients with RA and IBD and their matched controls with respect to diabetes, cancer, CVDs (eg, stroke, angina, myocardial infarction), depression and chronic kidney disease.

### Statistical analysis

Baseline characteristics between patients with RA and IBD and their matched controls were compared using descriptive statistics. Differences in the assessment of traditional CV risk factors and relevant treatment were compared between patients with RA and IBD with their matched controls without accounting for matching. These comparisons were calculated for the 12 months before, the 12 months following, as well as within 5 years from the case index date. Graphics were used to illustrate changes over time in the percentage of patients with measurements for traditional CV risk factors from 12 months before baseline to 5 years after the case index date. Conditional Poisson regression analysis was used to estimate differences cases and controls in the number of traditional CV risk factors assessed at the three specified time points. Conditional logistic regression was used to estimate differences in individual CV risk factors assessment and treatment at the prespecified time points. These analyses adjusted for age, gender, ethnicity and deprivation. Mixed-effects regression was used to estimate change over time in CV risk factors recording. Continuous QRISK scores were calculated and categorised into percentiles of 0%–10%, 10%–20% and >20%. All statistical analyses were performed using STATA V.14. An α level of 0.05 was used as the criterion for statistical significance.

## Results

The study identified 1121 patients diagnosed with RA who were matched with 4282 controls, and 1875 patients diagnosed with IBD and matched with 7308 controls (table 1). Among the patients with RA, 77% were female and had a mean age at RA diagnosis of 54 years. Among patients with IBD, 50% were female and the mean age at IBD diagnosis was 38 years. With respect to ethnicity, over a third were of white ethnic origins, about a sixth were of mixed ethnic origins and about a tenth of black origins. Just over 80% of both cases and controls were in the two most deprived quintiles. There were no major differences between cases and their matched controls with regard to chronic illnesses at baseline, with the exception of cancer, which was twice as common among RA matched controls (9%) compared with RA cases (4%). Overall, patients with RA had higher rates of chronic illnesses compared with patients with IBD.

**Table 1:** Characteristics of study participants at baseline

	RA patients (N=1121)	RA controls (N=4282)	IBD patients (N=1875)	IBD controls (N=7308)
Females	939 (77)	3741 (77)	943 (50%)	3677 (50%)
Age at diagnosis—mean (SD)	54 (15)	54 (15)	38 (19)	38 (19)
Ethnicity				
White	423 (35)	1677 (35)	780 (42)	2458 (33)
Black	165 (14)	732 (15)	181 (10)	1046 (15)
Asian	92 (8)	285 (6)	85 (4)	392 (5)
Mixed	233 (19)	870 (18)	446 (24)	1299 (18)
Other	158 (13)	539 (11)	99 (5)	565 (8)
Missing	146 (12)	747 (15)	286 (15)	1548 (21)
IMD quintile				
Most	461 (38)	1694 (35)	604 (32)	2505 (34)
Second	556 (46)	2340 (48)	93 6 (50)	3446 (47)
Third	150 (12)	573 (12)	233 (13)	932 (13)
Fourth	26 (2)	132 (3)	60 (3)	212 (3)
Least	/	5 (0)	/	4 (0.1)
Missing	24 (2)	106 (2)	42 (2)	209 (3)
Chronic kidney disease	39 (3)	146 (3)	28 (2)	92 (1)
Diabetes	134 (12)	469 (11)	125 (7)	431 (6)
Antihypertensive drugs*	74 (7)	341 (8)	60 (3)	265 (4)
Lipid-lowering drugs†	26 (2)	88 (2)	21 (1)	57 (1)
Cardiovascular disease	98 (9)	362 (8)	103 (5)	326 (4)
Depression	163 (15)	651 (15)	205 (11)	860 (12)
Cancer	48 (4)	397 (9)	54 (3)	229 (3)

Figures are frequencies and percentages, unless otherwise specified.

Percentages based on recorded data.

*Percentage of participants prescribed antihypertensive drugs in the year preceding disease diagnosis.

†Percentage of participants prescribed lipid-lowering drugs in the year preceding disease diagnosis.

IBD, inflammatory bowel disease; IMD, Index of multiple deprivation; RA, rheumatoid arthritis.

**Figure 1:**
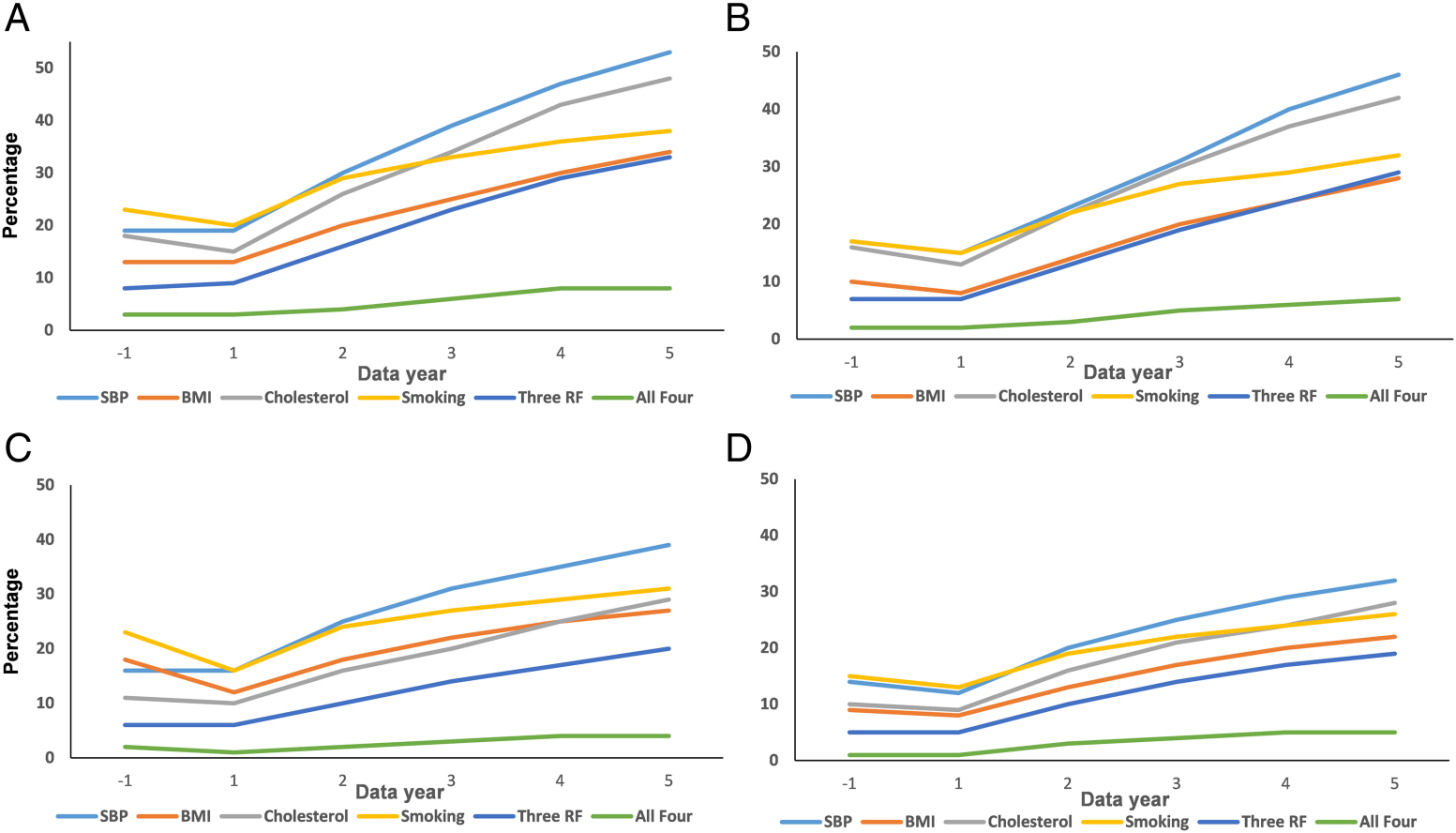
Cumulative trends in recording of vascular risk factors from the year before and up to 5 years following disease diagnosis in patients and controls. (A) Patients with rheumatoid arthritis. (B) Controls with rheumatoid arthritis. (C) Patients with inflammatory bowel disease. (D) Controls with inflammatory bowel disease. BMI, body mass index; RF, risk factors; SBP, systolic blood pressure.

Figure 1 illustrates the cumulative trends in CV risk factor recording from the 12 months before the case index date and up to 5 years following the case index date. There was no apparent change in the percentage of patients with RA or IBD with CV risk factors measured from the 12 months before to the 12 months after the index date. Cumulatively, the percentage of patients with IBD and RA with CV risk factors assessed increased substantively and was most commonly performed with regard to SBP and less commonly for BMI. At most a fifth of patients with RA (20%) and at most a sixth (16%) of patients with IBD had one CV risk factor measured within the first 12 months following the index date. These rates increased at 5 years of follow-up to about half (53%) among patients with RA and just over a third (39%) among patients with IBD. However, less than a tenth of patients with RA (8%) or IBD (4%) had all four CV risk factors recorded during the 5-year follow-up period. Time-trend analyses for all risk factors were statistically significant (p<0.001). Similar trends but of lower magnitude were observed among RA and IBD matched controls.

Table 2 illustrates that about a tenth of patients with RA and IBD had their BP, total cholesterol and BMI recorded during the 12 months following the index date, and that these measurement rates increased about threefold during the 5-year follow-up period. Notably, only a minority of patients with RA (3%) and IBD (2%) had their CVD risk score measured within the 12-month period following RA and IBD index date. These figures increased to about 11% (RA) and 6% (IBD) during the 5-year follow-up period. No significant differences between patients with RA or IBD and their matched controls were revealed with regard to CV risk scoring measurement. Compared with their matched controls, patients with RA were more likely to have SBP, smoking and BMI (with the exception of the 5 years of follow-up) measured following the index date. Similar findings emerged among patients with IBD. The proportion of patients with RA who were prescribed antihypertensive and lipid-lowering therapies at 12-month and 5-year follow-up period was 10% and 24%, and 5% and 15%, respectively. These figures were lower for non-RA matched controls (7% and 9%, and 5% and 13%, respectively). The difference reached statistical significance only with respect to antihypertensive therapy prescribing over the 5-year follow-up period (OR 1.40, 95% CI 1.12 to 1.75). Patients with IBD presented higher rates of lipid-lowering therapy (3.21, 1.44 to 7.15) before the index date, but not post-IBD diagnosis.

**Table 2:** Differences in the percentage of patients and controls with CV risk assessed and treated over time

	RA patients			RA controls			ORs (95% CI)		
	1 year before	1 year after	5 years after	1 year before	1 year after	5 years after	1 year before	1 year after	5 years after
BP recorded	130 (12)	125 (11)	347 (31)	427 (10)	385 (9)	1188 (28)	1.14 (0.89 to 1.46)	1.50 (1.10 to 1.90)	1.40 (1.15 to 1.70)
Hypertension*	329 (29)	472 (43)	545 (49)	1085 (26)	1130 (26)	1728 (40)	1.07 (0.59 to 1.94)	1.99 (1.20 to 3.28)	1.28 (0.94 to 1.76)
AHT	39 (3)	107 (10)	268 (24)	135 (3)	307 (7)	808 (19)	0.93 (0.60 to 1.47)	1.32 (0.94 to 1.85)	1.40 (1.12 to 1.75)
TC recorded	150 (13)	128 (11)	390 (35)	473 (11)	406 (9)	1253 (29)	1.34 (1.05 to 1.72)	1.15 (0.88 to 1.49)	1.21 (0.98 to 1.48)
High cholesterol†	59 (5)	50 (4)	148 (13)	206 (5)	173 (4)	578 (13)	1.19 (0.83 to 1.69)	0.98 (0.67 to 1.43)	0.83 (0.65 to 1.05)
LLD recorded	18 (2)	55 (5)	167 (15)	55 (1)	203 (5)	556 (13)	1.33 (0.68 to 2.65)	1.02 (0.68 to 1.55)	1.20 (0.93 to 1.55)
Smoking recorded	257 (23)	235 (21)	427 (38)	736 (17)	664 (15)	1346 (32)	1.57 (1.29 to 1.90)	1.44 (1.17 to 1.77)	1.25 (1.05 to 1.50)
Current smoker	149 (13)	127 (11)	205 (18)	341 (8)	317 (7)	591 (14)	1.88 (1.48 to 2.38)	1.69 (1.30 to 2.20)	1.45 (1.17 to 1.80)
BMI recorded	150 (13)	139 (13)	374 (34)	436 (10)	351 (8)	1226 (28)	1.52 (1.19 to 1.93)	1.49 (1.15 to 1.92)	1.21 (0.99 to 1.48)
Obesity‡	50 (4)	54 (5)	144 (13)	163 (4)	120 (3)	486 (11)	1.38 (0.94 to 2.00)	1.64 (1.12 to 2.40)	1.06 (0.83 to 1.34)
Diabetes mellitus	88 (8)	120 (11)	270 (24)	279 (7)	323 (8)	846 (20)	1.30 (0.94 to 1.80)	1.42 (1.07 to 1.89)	1.22 (0.99 to 1.48)
CVD risk scores§	31 (3)	28 (2)	121 (11)	144 (3)	143 (3)	479 (11)	0.72 (0.46 to 1.14)	0.79 (0.50 to 1.26)	0.91 (0.70 to 1.18)
QRISK score data¶	45 (4)	22 (2)	191 (17)	129 (3)	86 (2)	685 (16)	1.42 (0.76 to 2.68)	0.93 (0.84 to 4.46)	1.22 (0.91 to 1.63)
	**IBD patients**			**IBD controls**			**ORs (95% CI)**		
	**1 year before**	**1 year after**	**5 years after**	**1 year before**	**1 year after**	**5 years after**	**1 year before**	**1 year after**	**5 years after**
BP recorded	248 (13)	234 (12)	520 (28)	716 (10)	620 (8)	1662 (23)	1.36 (1.13 to 1.64)	1.58 (1.30 to 1.91)	1.37 (1.16 to 1.61)
Hypertension	246 (14)	362 (19)	430 (23)	977 (14)	957 (13)	1440 (20)	1.77 (0.96 to 3.24)	2.07 (0.99 to 4.32)	1.30 (0.88 to 1.92)
AHT	32 (4)	77 (7)	213 (11)	112 (2)	287 (4)	730 (10)	1.16 (0.72 to 1.87)	1.12 (0.78 to 1.62)	1.19 (0.94 to 1.49)
TC recorded	206 (11)	177 (10)	549 (29)	672 (10)	648 (9)	2026 (28)	1.27 (1.01 to 1.60)	1.09 (0.86 to 1.39)	1.13 (0.94 to 1.34)
High cholesterol	61 (3)	47 (3)	154 (8)	190 (3)	203 (3)	648 (9)	1.23 (0.88 to 1.74)	0.82 (0.56 to 1.120)	0.79 (0.63 to 0.98)
LLD recorded	16 (1)	47 (3)	142 (8)	26 (0)	175 (2)	440 (7)	3.21 (1.44 to 7.15)	0.98 (0.63 to 1.54)	1.05 (0.80 to 1.36)
Smoking recorded	422 (23)	319 (17)	576 (31)	1113 (15)	903 (13)	1920 (26)	1.49 (1.27 to 1.79)	1.32 (1.11 to 1.57)	1.16 (0.99 to 1.34)
Current smoker	183 (10)	146 (8)	255 (14)	590 (8)	490 (7)	942 (13)	1.17 (0.95 to 1.43)	1.12 (0.89 to 1.40)	0.98 (0.81 to 1.18)
BMI recorded	332 (18)	235 (12)	520 (27)	720 (10)	573 (8)	1606 (22)	2.24 (1.87 to 2.68)	1.61 (1.32 to 1.96)	1.31 (1.12 to 1.53)
Obesity	40 (2)	41 (2)	119 (6)	197 (3)	220 (3)	571 (8)	0.80 (0.54 to 1.18)	0.72 (0.48 to 1.06)	0.77 (0.60 to 0.98)
Diabetes mellitus	73 (4)	115 (6)	255 (14)	245 (3)	365 (5)	883 (12)	1.20 (0.84 to 1.71)	1.28 (0.97 to 1.61)	1.14 (0.94 to 1.39)
CVD risk scores	33 (2)	32 (2)	111 (6)	156 (2)	123 (2)	430 (6)	0.71 (0.46 to 1.10)	0.90 (0.56 to 1.44)	0.90 (0.72 to 1.22)
QRISK score data	123 (11)	35 (2)	142 (17)	514 (12)	162 (2)	542 (16)	0.81 (0.41 to 1.59)	1.47 (0.81 to 2.68)	1.19 (0.90 to 1.56)

Figures are frequencies and percentages, unless otherwise stated.

*Hypertension defined as systolic blood pressure ≥140 mm Hg or diastolic blood pressure ≥90 mm Hg.

†High cholesterol defined as total cholesterol ≥5.0 mmol/L.

‡Obesity defined as BMI ≥30 kg/m^2^.

§Risk score recorded by practitioners.

¶QRISK scores in the year before diagnosis defined as between registration and diagnosis.

AHT, antihypertensive drugs; BMI, body mass index; BP, blood pressure; CVD, cardiovascular disease; IBD, inflammatory bowel disease; LLD, lipid-lowering drugs; RA, rheumatoid arthritis; TC, total cholesterol.

Patients with RA were more likely to have hypertension (1.99, 1.20 to 3.28), be classified as obese (1.64, 1.12 to 2.40) at 12 months of follow-up and be current smokers at all time points compared with their matched controls. Patients with IBD presented similar association patterns, with the exception of BMI where no evidence of significant differences in assessment was evident. The only significant differences in diabetes rates were observed among patients with RA at 12 months of follow-up (1.42, 1.07 to 1.89).

Conditional Poisson regression analyses (table 3) revealed significantly higher rates of CV risk factors measurement across the three time points among patients with both RA and IBD. For instance, at 12 months of follow-up, patients with IBD were 26% (incidence rate ratio, 1.26, 95% CI 1.16 to 1.38) more likely to have one or more CV risk factors measurement compared with their matched controls. Similar association but of lower magnitude (1.10, 1.05 to 1.15) was observed during the 5-year follow-up period.

**Table 3:** Conditional Poisson regression analysis for differences in CV risk factor measurement among patients with RA and IBD compared with their matched controls

	RA	N (%)	IBD	N (%)
Time period				
1 year before index date	1.22 (1.11 to 1.33)	3171 (59)	1.33 (1.23 to 1.44)	4840 (53)
1 year after index date	1.25 (1.12 to 1.35)	3200 (59)	1.26 (1.16 to 1.38)	4428 (48)
Within 5 years from index date	1.08 (1.04 to 1.14)	3941 (73)	1.10 (1.05 to 1.15)	5473 (60)

The incidence rate ratios were adjusted for ethnicity, age, gender and deprivation.

CV, cardiovascular; IBD, inflammatory bowel disease; N, number; RA, rheumatoid arthritis.

## Discussion

This study represents one of the few prospective studies on the assessment and treatment of CV risk factors among an ethnically diverse and deprived RA and IBD primary care population. The study compared rates and mean values of CV risk factor as well as CV treatment in the 12 months before disease diagnosis, within 12 months from disease diagnosis, and subsequently and up to 5 years following disease diagnosis. Overall the evidence implies suboptimal assessment of CVD risk among two of the most common inflammatory disorders. While patients with RA and IBD were more likely to have CV risk factors measured and treated compared with their matched controls, this association occurred in the context of very low rates of CV risk factors assessment and treatment. Just about a tenth of patients with RA had all four CV risk factors measured—essential for accurate estimation of future CVD risk—in the 12 months following disease diagnosis. The same figure for patients with IBD stood at just 6%, documenting important discrepancies within the same group of disorders. While there was encouraging evidence for improved rates of CV risk factors measurement and treatment in the long term, our study data enabled the calculation of a CVD risk score at 5 years of follow-up for about a fifth of patients with RA and IBD. When comparing the two inflammatory conditions, patients with RA presented higher rates of CV risk factors measurement and treatment relative to patients with IBD. This difference may be explained by the availability of National Institute for Health and Care Excellence recommendations for CVD risk screening among patients with RA newly diagnosed, while general recommendations for CVD risk screening are missing for patients with IBD. Or by the greater availability of empirical evidence for increased CVD risk among patients with RA compared with patients with IBD.

Given that primary care practitioners are responsible for CVD prevention in the general population and the award of financial incentives for complete CV risk monitoring, higher rates of CV risk factors measurement and treatment were expected. Several factors may account for the low rates of CV risk factors measurement and treatment observed in this study. Some practitioners may use a medical code denoting hypertension or obesity without recording the actual value, rendering the information of little value when trying to estimate a CVD risk score. The younger mean age (38 years) at IBD diagnosis means that it may be uncommon to observe high rates of CV risk factors (ie, SBP, hypercholesterolaemia) in this age group and thus practitioners may be less likely to screen these patients for the presence of CV risk factors. Besides, existing CVD risk score, instruments (eg, Reynolds CVD risk, QRISK II, SCORE) tend to underestimate future CVD events among patients with RA possibly disincentivising practitioners from recording CV risk factors data. Also, since patients with both RA and IBD would be under the care of a specialist (ie, rheumatologist, gastroenterologist), there may be the expectation that CV risk would also be assessed in specialist clinics. Future studies that combine data from both primary and secondary care are needed to validate this suggestion and also to understand the extent to which CV risk screening is shared or not among different clinicians. In addition, methodological shortcomings of the study data (eg, under-reporting, lower proportion of people over 65 years of age) may also partially account for the study findings.

Risk charts used for CVD risk stratification in patients with rheumatic diseases, in particular in those with RA, have proved to underestimate the actual CVD risk of these patients. It is especially true for those included in the category of moderate (intermediate) risk. With respect to this, a cohort study disclosed that 63% of patients with RA classified as having moderate CVD risk according to the SCORE had severe carotid ultrasound abnormalities (mainly carotid plaques).^12^ Therefore, besides traditional algorithms that should be routinely used in all cases, additional tools should be considered for CVD risk assessment of patients with inflammatory diseases.^12^ ^13^

Despite a breadth of evidence as to the association of chronic inflammatory (CI) disorders with increased risk of CVD,^5^ ^14^ there is limited evidence in the current literature about CV risk factors assessment and treatment in routine care. Alemao *et al*^10^ found no substantial difference in the assessment, treatment and attainment of CV risk factor goals among a representative sample of patients with RA and non-RA matched controls. Similarly, Monk *et al*^15^ identified similar rates of CVD screening among patients with RA and age-matched, gender-matched and practice-matched non-RA patients. Our study findings for significant differences in assessment and treatment of CV risk factors between patients with RA and matched controls imply that at a more local-level important differences may be observed. The Lambeth DataNet is a local dataset and it may have picked up on differences that nationwide datasets^10^ may have overlooked. Our study findings are in line, however, with recent European and US-based evidence.^16–18^

### Strengths and limitations

Our study has several important strengths including prospective design, while cases and control groups were selected from the same population and information was collected in the same manner; thus, selection and information bias is likely to be minimal. Patients were included only if they had been registered at the practice for 12 months or more, ensuring accurate date of diagnosis and reliable clinical and therapy data. Our data source has a high level of ethnicity recording and reflects patients from a very wide range of socioeconomic backgrounds, promoting the generalisability of the findings. However, as with any observational data there are important limitations that need acknowledgement. We drew on the READ code classification, used in UK primary care to identify chronic inflammatory disease diagnosis, comorbidities, treatment and risk factor management. We acknowledge that alternative codes might be proposed. Only data from primary care setting were available and no data from specialist clinics were available, which could impact the identification of patients and CV risk factors assessment into our study. Confounding is also common in observational studies and there may be other relevant factors that were unavailable to us. Also, primary care practitioners may record some of the CV risk factors data in a textual format, which was not available to us. It is also possible that primary care practitioners may not record CV risk factors information collected previously if there was no apparent change in these factors, particularly with respect to smoking and BMI. While this suggestion may partially explain the low rates of measurement for some CV risk factors, it is unlikely to be responsible for the low rates observed among SBP and cholesterol levels. The poor predictive value of existing risk scores may also discourage their use in inflammatory patients.

## Conclusion

On the positive side, our study findings document greater rates of SBP, BMI and smoking assessment among patients with RA and IBD compared with their matched controls. Unfortunately, this finding was outdone by the substantively low rates of CV risk factors measurement and treatment among a group of patients known to be at heightened risk of future CVD events. Of particular concern was that a CVD risk score calculation was possible for only 2% of the patients with RA and IBD within the first 12 months following disease diagnosis. Even when considered individually and over a 5-year period, less than half of patients with RA and IBD had their CV risk factors assessed. Our study findings emphasise the need for initiatives to increase primary care practitioners' awareness about the importance for recording data on CV risk factors among patients with RA and IBD. Given the limited feasibility for this information to be collected within the constraints of a time-limited consultation, alternative strategies for CV risk factors data collection need to be tested (ie, patient-reported data, nurse-based assessment, improved primary–secondary care communication).

**Key messages**

**What is already known on this subject?**

- Patients diagnosed with chronic inflammatory disorders are at increased risk of cardiovascular disease (CVD) events. Existing evidence documented poor CV risk factors assessment and management in rheumatology clinics. Less evidence is available, however, from primary care practices and across different inflammatory disorders.

**What might this study add?**

- CV risk assessment and management in primary care practices is very low. Patients with inflammatory bowel disease (IBD) have lower rates of CV risk factors assessment and management comparing to their rheumatoid arthritis (RA) counterparts. Only a small minority of patients with RA and IBD are assessed for the presence of all four CV risk factors to enable calculation of a CV risk score

**How might this impact on clinical practice?**

- Currently, only a minority of patients with RA and IBD are assessed for the presence of CV risk factors in primary care settings. Primary care practitioners should be encouraged and supported to screen for CV risk among patients with RA and IBD.
